# A209 EVALUATION OF THE ROLE OF DUSP1 AND DUSP16 GENES IN THE REGULATION OF INTESTINAL EPITHELIAL FONCTIONS

**DOI:** 10.1093/jcag/gwae059.209

**Published:** 2025-02-10

**Authors:** R Huard, J C Ntunzwenimana, C Lévesque, M Rivard, P Goyette, J D Rioux

**Affiliations:** Universite de Montreal, Montreal, QC, Canada; Institut De Cardiologie de Montreal, Montreal, QC, Canada; Institut De Cardiologie de Montreal, Montreal, QC, Canada; Institut De Cardiologie de Montreal, Montreal, QC, Canada; Institut De Cardiologie de Montreal, Montreal, QC, Canada; Universite de Montreal, Montreal, QC, Canada

## Abstract

**Background:**

Inflammatory bowel disease (IBD) is associated with inflammation of the gastrointestinal tract, resulting from a dysregulation of the immune response to microbes in the intestinal lumen. Dr. Rioux’s laboratory has identified over 200 genomic regions associated with IBD. However, the challenge lies in identifying the causal gene in many of these. To address this problem, our laboratory completed a transcriptomic screen following overexpression of IBD-associated genes in intestinal epithelial cells. This study demonstrates that DUSP1 and DUSP16, two phosphatases targeting MAP Kinases (MAPKs), regulate the expression of several shared genes involved in intestinal differentiation and homeostasis.

**Aims:**

Our research hypothesis is that the DUSP1 and DUSP16 genes participate in the regulation of biological processes involved in IBD and critical for the integrity of the intestinal barrier. More specifically, we want to determine how a reduction in DUSP1 and DUSP16 affects the biological pathways leading to the regulation of epithelial junction integrity, as well as inflammasome activation and IL-18 secretion.

**Methods:**

In this project, we studied Caco-2 cells expressing DUSP1 or DUSP16 knockdown and their effect on intestinal epithelial cell functions. Among other things, we observed the cells’ ability to form a tight monolayer in 2D and spheroids in 3D. We also analyzed by Western blotting the impact of knockdowns on inflammasome formation and normal activity.

**Results:**

Our results show that Dusp1 and DUSP16 knockdowns disrupt the ability of Caco-2 cells to form a tight monolayer and to form spheroids. In addition, our inflammasome analysis shows that KDs deregulate inflammasome activity.

**Conclusions:**

These results demonstrate that decreased expression of the DUSP1 and DUSP16 genes impacts several functions essential to the maintenance of intestinal epithelial cell homeostasis, such as intestinal barrier integrity and inflammasome activation.

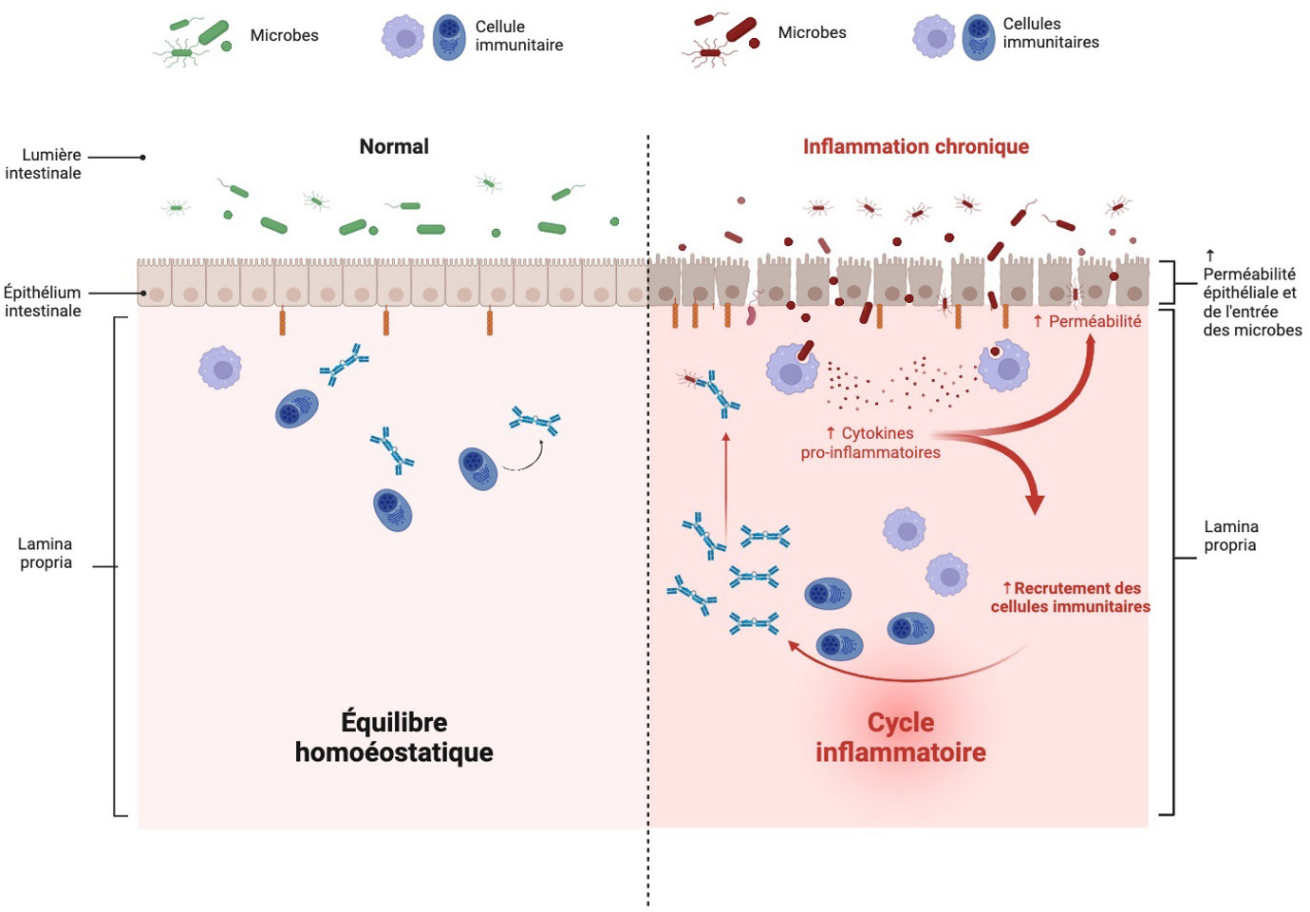

**Funding Agencies:**

CIHRNIH

